# Complete Genome Sequence of an *mcr-10*-Carrying Enterobacter roggenkampii Strain Isolated from a Human Blood Culture

**DOI:** 10.1128/mra.00075-23

**Published:** 2023-03-13

**Authors:** Chengcheng Wang, Yu Feng, Zhiyong Zong

**Affiliations:** a Center of Infectious Diseases, West China Hospital, Sichuan University, Chengdu, China; b Center for Pathogen Research, West China Hospital, Sichuan University, Chengdu, China; c Division of Infectious Diseases, State Key Laboratory of Biotherapy, Chengdu, China; University of Maryland School of Medicine

## Abstract

The *mcr-10* gene is a plasmid-borne colistin resistance gene. The complete genome sequence of an *mcr-10*-carrying Enterobacter roggenkampii clinical strain that had been isolated from a blood culture was obtained. The genome consists of a 4.95-Mbp chromosome and six plasmids, including a 195-kb plasmid harboring *mcr-10* and two replicons, IncFIB(K) and IncFII(Yp).

## ANNOUNCEMENT

Mobile colistin resistance (*mcr*) genes, including *mcr-1* to *mcr-10*, represent an emerging challenge for antimicrobial therapy (1–3). The *mcr-10* gene was first identified on an 71,775-bp IncFIA(HI1) plasmid of an Enterobacter roggenkampii strain that had been recovered from ascites in 2020 (2). Here, we describe the genome sequence of another *mcr-10*-carrying E. roggenkampii strain.

Strain 120063 was recovered from blood from a febrile patient at West China Hospital in 2019. This study was approved by the ethics committee of the hospital, with the requirement for informed consent being waived. This strain was recovered from a BacT/Alert blood culture bottle (bioMérieux, Durham, NC) after a 24-h aerobic incubation at 37°C. The strain was then inoculated on Luria-Bertani agar at 37°C for 24 h. Single colonies were used for antimicrobial susceptibility testing and DNA extraction. Strain 120063 was resistant to colistin (MIC, 4 mg/L), as determined using the CLSI broth microdilution method (4).

Genomic DNA for short-read sequencing was extracted using the QIAamp DNA blood minikit (Qiagen, Hilden, Germany), and libraries were prepared using the NEBNext Ultra II kit (New England Biolabs, Ipswich, MA). Sequencing (150-bp paired-end reads) was performed on the HiSeq X Ten platform (Illumina, San Diego, CA). Reads were trimmed 10 bp from both ends with Cutadapt v4.0 (5). Adapter removal and quality filtering were performed using BBMap v39.01 (https://jgi.doe.gov/data-and-tools/software-tools/bbtools) (minimum quality, Q15; minimum length, 50 bp). There were 11,405,928 short reads (average length, 150 bp).

For long-read sequencing, genomic DNA was prepared from fresh colonies using the Monarch genomic DNA purification kit (New England Biolabs) and was not sheared or size selected but was cleaned using AMPure XP beads (1:1) (Beckman Coulter, Brea, CA). Libraries were prepared with multiplexing using the rapid barcoding kit (Oxford Nanopore Technologies, Oxford, UK) and were sequenced on a MinION system with an R9.4.1 flow cell (Oxford Nanopore Technologies). Base calling and demultiplexing were performed using Guppy v4.4.0 with config file dna_r9.4.1_450bps_hac.cfg. Quality control was performed using NanoFilt v2.8.0 (6) (minimum quality, Q8; minimum length, 1,000 bp), which generated 262,017 long reads (read *N*_50_, 12,754 bp). Trimmed long reads were fed into assemblers Canu v2.2 (7), Flye v2.9.1 (8), miniasm v0.3_r179 (9) with minipolish v0.1.3 (10), and Raven v1.8.1 (11), generating multiple complete assemblies of the same genome. Trycycler v0.5.3 (12) clustered contigs from different assemblies, producing a consensus contig for each cluster. These consensus contigs were polished with Medaka v1.7.2 (https://github.com/nanoporetech/medaka/releases), Polypolish v0.5.0 (13), and MaSuRCA v4.1.0 (14) in turn and combined into a final high-quality assembly. Default parameters were used for all software unless otherwise specified.

NCBI Prokaryotic Genome Annotation Pipeline (PGAP) v6.4 (15) was used for annotation. Precise species identification was established based on the average nucleotide identity (ANI) between strain 120063 and type strains of Enterobacter species with JSpeciesWS (16). Antimicrobial resistance genes and plasmid replicons were identified from genome sequences using abricate v1.0.1 (https://github.com/tseemann/abricate) to query ResFinder (17) and PlasmidFinder (18), respectively.

**FIG 1:**
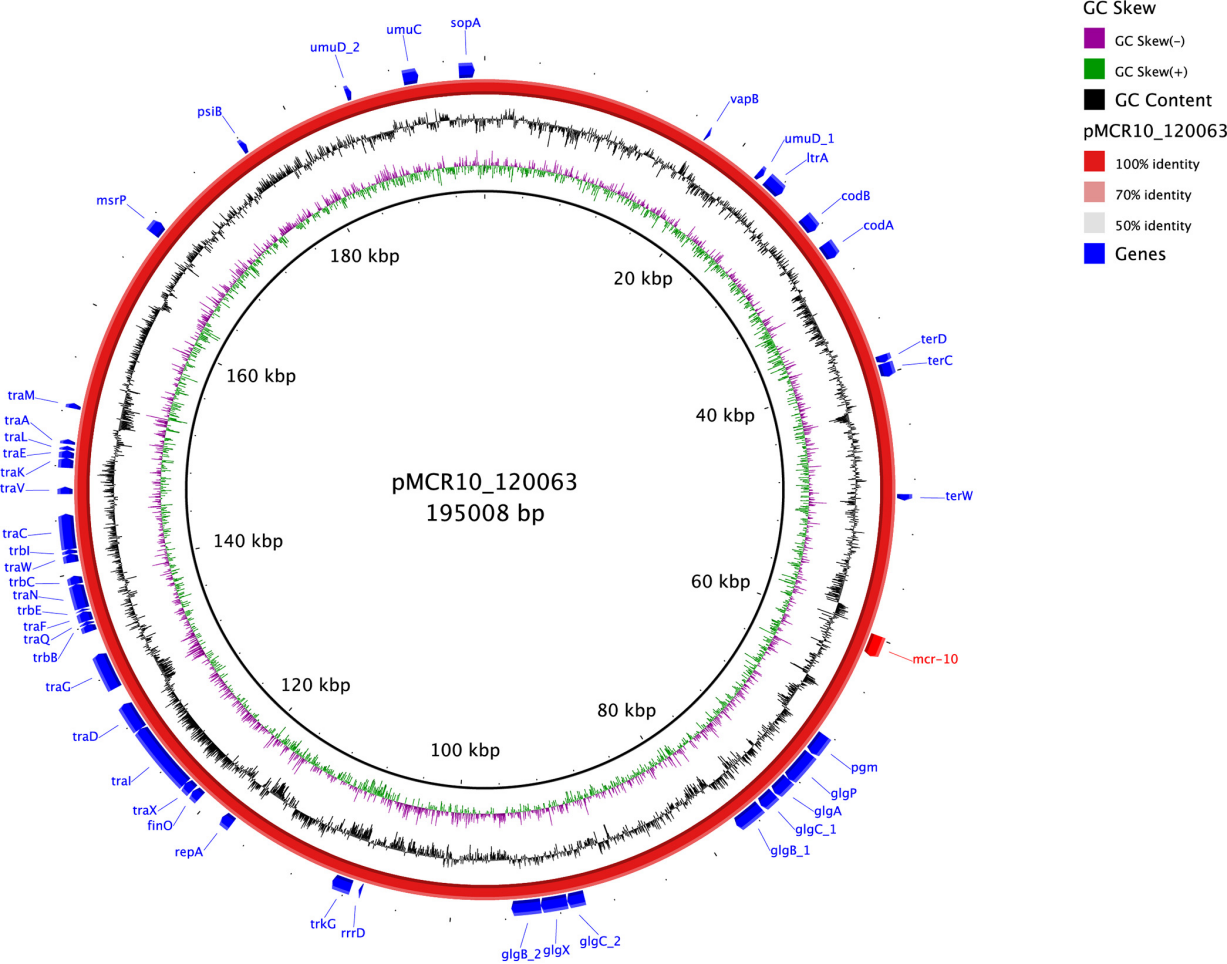
Circular map of the complete *mcr-10*-carrying plasmid pMCR10_120063. The *mcr-10* gene is labeled in red, and other genes are labeled in blue. This circular map was generated using BLAST Ring Image Generator (BRIG) software. Detailed information about pMCR10_120063 is available under GenBank accession number CP116250.

The complete genome of 120063 comprises a circular 4,948,818-bp chromosome and six circular plasmids. Strain 120063 belongs to E. roggenkampii, sharing 98.54% ANI with E. roggenkampii DSM16690^T^ (GenBank accession number CP017184), and *mcr-10* was located on plasmid pMCR10_120063 (195,008 bp [IncFIB(K) and IncFII(Yp)]) (Fig. 1).

### Data availability.

The complete genome sequence of 120063 was deposited in GenBank under the accession numbers CP116249 to CP116255 and BioProject accession number PRJNA415108. The Illumina and MinION sequence reads were deposited in the Sequence Read Archive (SRA) database under accession numbers SRX19039017 and SRX19041193, respectively.
